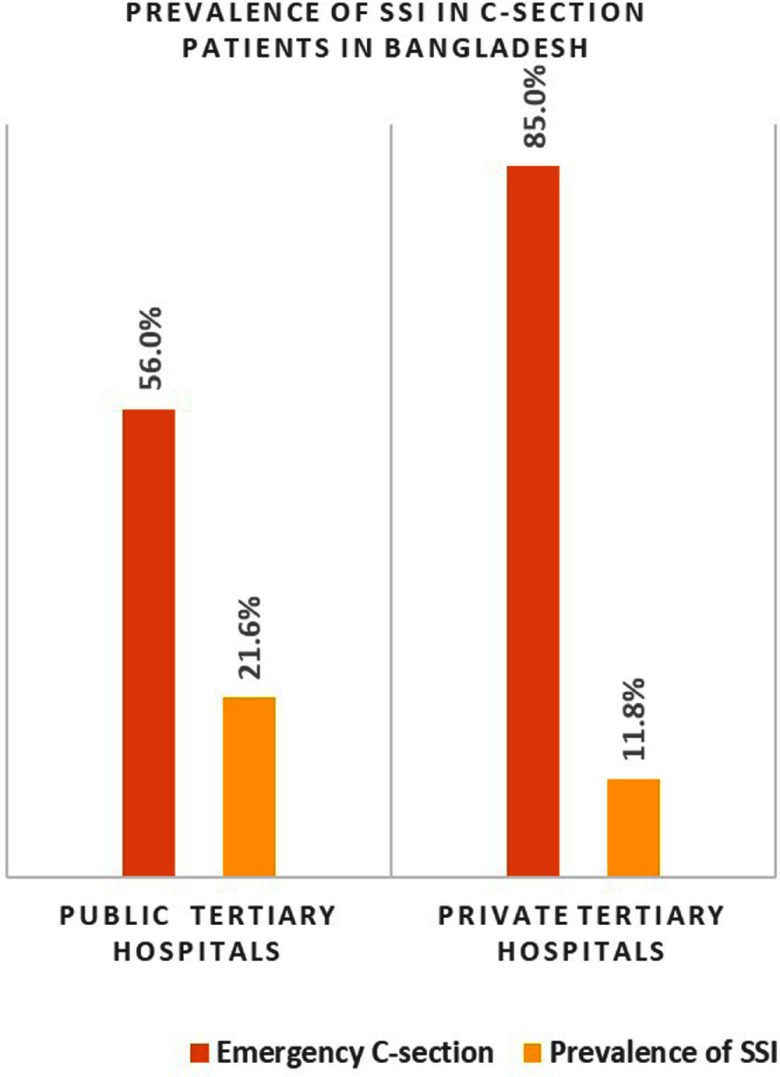# Surgical site infections among cesarean patients in Bangladeshi hospitals: results from an observational study

**DOI:** 10.1017/ash.2025.405

**Published:** 2025-09-24

**Authors:** Shariful Amin Sumon, Aninda Rahman, Syed Abul Hassan Md Abdullah, Md. Golam Dostogir Harun

**Affiliations:** 1 icddr,b; 2 Communicable Disease Control, MOHFW; 3 South Asia Field Epidemiology & Technology Network (Safetynet); 4 International Centre for Diarrhoeal Disease Research, Bangladesh (Icddr,b)

## Abstract

**Background:** Surgical site infections (SSIs) following cesarean deliveries (C-sections) result in excess morbidity, mortality, and healthcare expenses in resource-limited countries such as Bangladesh. Over the past two decades, C-section rates have increased dramatically in Bangladeshi hospitals, and comprehensive data on SSI after cesarean delivery, which is vital for the improvement of maternal health outcomes, remains limited. In this study, we assessed the prevalence of SSIs including their determinants among patients undergoing C-sections in Bangladesh. **Methods:** From May to December 2023, we conducted a prospective observational study at six tertiary hospitals (3 public and 3 private) in Bangladesh. Participants were hospitalized pregnant women who had undergone C-sections. The WHO-guided methodology and tools were employed to acquire the data. Participants were systematically evaluated on days 1-3, 7, 14, and 30 of surgeries, with a rigorous inquiry into symptoms such as fever, abdominal pain, localized swelling and redness, wound dehiscence, and purulence or abscess. The SSI diagnosis was confirmed based on at least two present symptoms, or a physician’s assessment, or microbiological confirmation within the 30-day post-operative window. Descriptive and multivariate logistic analyses were performed to determine the prevalence and factors associated with SSI. **Results:** Of 1335 participants enrolled, the overall prevalence of SSIs was 19.1% (255/1335, 95%CI: 17.6-21.5), with public hospitals having almost twice as SSIs at 21.6% (215/995) compared to private hospitals (11.8%, 40/340). More than half of the patients (54.8%) were found with at least two SSI symptoms within the 7 to 14 days of follow-up. Approximately half of the patients (49.2%) had a history of previous C-sections. The C-sections performed in private hospitals were predominantly on an emergency basis (85.1%) compared to public hospitals (56.2%). The multivariate analysis identified key determinants of SSI following C-section were patients with prolonged labor > 18 hours (AOR: 2.2, 95%Cl: 1.16, 4.13), fetal distress (AOR: 1.82, 95%Cl: 1.33, 2.49), premature rupture of membrane (PROM) > 12 hours (AOR: 1.70, 95%Cl: 1.05, 2.75), and high BMI (AOR: 1.69, 95%Cl: 1.27, 2.25). **Conclusions:** This study highlights the burden of SSIs following C-sections in tertiary hospitals in Bangladesh, particularly in public healthcare settings. The findings highlight the critical need to enhance infection prevention and control measures to mitigate the occurance of SSIs within these healthcare settings.

**Figure:**